# Early Onset Multiple Sclerosis Has Worse Prognosis Than Adult Onset Multiple Sclerosis Based on Cognition and Magnetic Resonance Imaging

**DOI:** 10.1155/2012/563989

**Published:** 2012-11-07

**Authors:** Serkan Ozakbas, Derya Kaya, Egemen Idiman

**Affiliations:** Department of Neurology, Dokuz Eylül University, Balçova, 35340 İzmir, Turkey

## Abstract

*Objectives*. In the present study, we aimed to compare the childhood and adult onset multiple sclerosis patients prospectively in their adulthood on the basis of clinical and magnetic resonance imaging (MRI) findings and cognitive impairment, which have not been performed before. *Patients and Methods*. Forty-six patients in whom the disease onset occurred before 16 years of age were included in the present study. Study subjects were compared with 64 randomly included adult onset patients. *Results*. Mean disease duration, clinical course, and female to male ratio did not differ in the groups. Cerebellar/brainstem and spinal involvement at onset were significantly higher in EOMS than in AOMS. Difference in MSFC between baseline and at the end of the 5th year was significantly worse in EOMS population (*P* = 0.02). The most significant difference was found in Paced Auditory Serial Addition Test (PASAT) (*P* = 0.008). Differences between baseline and at the end of the 5th year on the basis of T1 hypointense lesions were significantly higher in early onset MS than in adult onset MS patients (*P* = 0.02). *Conclusions*. Early onset MS seems to have worse prognosis than that of adult onset MS on the basis of clinical manifestation, cognitive impairment, and MRI parameters.

## 1. Introduction

In 1.2–6% of multiple sclerosis (MS) cases, the onset of disease is before 16 years of age [1–8]. The accumulation of disabilities and the development of secondary progressive MS most commonly occur more than 15 years after the first attack. Prognosis of the patients with low age of onset is one of the main questions in childhood and juvenile onset MS (early onset MS—EOMS). Some authors found a better prognosis for patients with low age of onset [2, 3, 5], other authors reported a more favorable prognosis for patients with higher age [9], and some of them could find no influence on the clinical features of age at onset [6]. Discussion about the existence of clinical courses different from that of adult onset MS (AOMS) is also still open. MS can cause both physical and cognitive disabilities. Cognitive impairment estimated in 40% to 65% of cases [10]. In these patients, the impact on cognitive functioning was hypothesized even more dramatic than that observed in adult onset cases, because of the development of inflammatory demyelinating process during primary CNS myelinogenesis [11]. Therefore, negative effects on ongoing maturation of white matter pathways may involve cognitive networks. Available information on neuropsychological aspects of childhood and juvenile MS has been rather limited [12, 13]. It is also difficult to generalize data from published studies because of a number of limitations, such as the lack of a suitable control group and availability of comparing with adult MS patients or the absence of a global measure of patient cognitive function [11].

There have been no longitudinal magnetic resonance imaging (MRI) studies in EOMS to establish progression on the basis of imaging and to compare these data with AOMS.

Most studies on EOMS either have been based on a small number of cases and have not allowed obtaining exhaustive information on its prognosis or have been compared to a previous retrospective study [14]. In the present study, we aimed to compare the childhood and adult onset multiple sclerosis patients prospectively in their adulthood on the basis of clinical and magnetic resonance imaging (MRI) findings and cognitive impairment, which have not been performed before.

## 2. Patients and Methods

### 2.1. Patients

A prospective cohort of 46 patients in whom the disease onset occurred before 16 years of age, diagnosed with MS according to the McDonald's diagnostic criteria [15] and observed for at least two years were included in the present study. All patients had experienced two or more attacks with objective clinical evidence of 2 or more central nervous lesions. Cases with Devic's disease are not included. The current age ranged from 19 to 27 years old with a mean of 24.1 years old. EOMS patients were compared with 64 adult onset patients who were matched based on sex and disease duration. Age at the time of onset, initial symptoms, time between the onset and first relapse, clinical courses, contents of treatment, complications, and prognoses were investigated in the medical records. Clinical course was classified according to the standardized definition reported by Lublin and Reingold [16]. Informed consent was obtained from each patient. Patients were assessed at baseline, in the second year and in the fifth year.

Family history and associated diseases were investigated in all MS patients. Twenty-three percent of AOMS and 24% of EOMS patients were under disease-modifying treatments at baseline. Only 1 EOMS patient and 2 AOMS patients were treated with azathioprine add-on therapy.

Those patients who showed only recurrent transverse myelopathy, recurrent optic neuritis, or recurrent brainstem symptoms were excluded. Patients who were serologically positive for rheumatoid factor, antidouble stranded DNA antibody, were also excluded. All of the patients were negative for anticardiolipin antibody (aCL) and antineutrophil cytoplasmic antibody (ANCA). Patients were screened for sarcoidosis.

### 2.2. EDSS and MSFC

Clinical assessment of patients was done with Expanded Disability Status Scale (EDSS) and multiple sclerosis functional composite (MSFC). The EDSS [17] is the most widely used instrument for evaluating patients with MS in clinical trials as a primary clinical outcome. There are some drawbacks to the EDSS. For example, at any range of the scale, cognition is poorly assessed, even though cognitive impairments are common in MS patients.

The MSFC, which has been shown to be very effective to demonstrate the disability change for treatment effect [18] and follow-up difference [19], includes quantitative tests of leg function (Timed 25-Foot Walk—T25W), arm function (9-Hole Peg Test-9—HPT), and cognitive function (Paced Auditory Serial Additional Test—PASAT—3 sec version). The MSFC was administered to patients as described in the standardized protocol [20].

### 2.3. Magnetic Resonance Imaging

Patients were also compared on the basis of magnetic resonance imaging (MRI) data. Diagnostic MRI had been performed for all patients at the time of the first clinical attack or during the clinical course, and serial MRI had been carried out in several patients to evaluate the disease activity. For MRI assessment, the same MRI machine with 1.5 T was used for both baseline and year five. T2 hyperintense lesions, Gd-enhanced lesions, and T1 hypointense lesions (black holes) were calculated.

### 2.4. Procedure

The same physician administered the MSFC to a given patient at each study visit. All examining technicians were trained neurologists, and each neurologist was blinded to the other results of tests, MRI findings or disease history. The physician assessing EDSS score of study group was blinded to control results. MRI lesions were also assessed by a physician who was blinded to clinical findings.

Patients on steroid treatment one month of the assessment were not included, and they were reassessed.

### 2.5. Analysis

When analyzing changes in the individual MSFC components, we used the change in *Z* score of T25WT, 9-HPT, and PASAT. Comparison between clinical and demographic variables was made by 2 × *κ* contingency tables *χ* ^2^ tests for categorical variables and Kruskal-Wallis *χ* ^2^ tests for continuous ones. Changes in the EDSS (ΔEDSS) score of the patients at baseline and at the end of the second and fifth year were assessed by Friedman's ANOVA. For analyzing changes on overall MSFC score, its components (ΔMSFC, ΔPASAT, Δ9-HPT, and Δ25-TWT), and number of T2-, T1- and Gd-enhanced lesions at baseline and the fifth year, repeated measurements ANOVA were performed.

## 3. Results

General demographic and clinical features were summarized in Table 1. In the EOMS group, mean disease duration was 8.54 years, and it was not significantly different from the AOMS group (7.97 years, *P* = 0.78). According to clinical course, most of the patients had RR course and there was no significant difference between the groups on the basis of course of the disease. The female/male ratio did not differ in the groups. Demographic and clinical features were summarized in the table. Cerebellar/brainstem and spinal involvement at onset were significantly higher in EOMS than AOMS (*P* = 0.005 and *P* = 0.02, resp.). The EDSS evaluated at baseline examination was not significantly different in both groups, although it was higher in AOMS than EOMS (see Table 1). In EOMS group, mean EDSS score worsened from 3.19 ± 2.71 at baseline to 3.65 ± 2.54 at year 2 (*P* = 0.02) and 4.06 ± 3.29 at year 5. AOMS group was also found to be worsened at year 2 and year 5, on the basis of EDSS (3.46 ± 1.9, 3.79 ± 1.9, and 4.21 ± 3.1, resp.).

**Figure 1:**
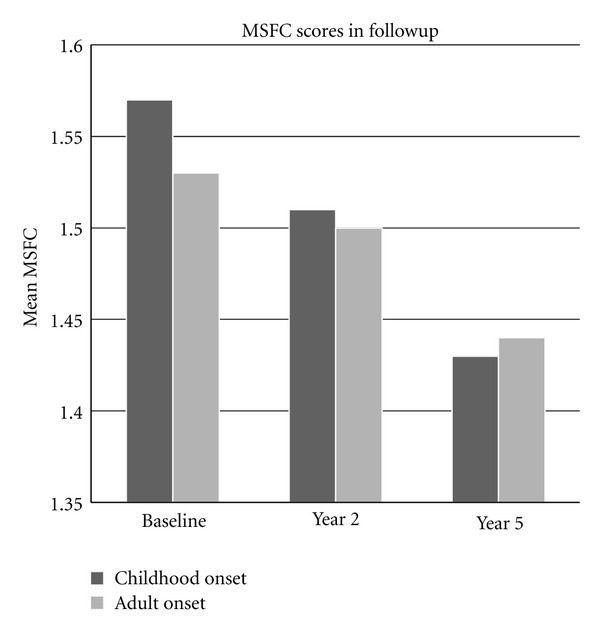
MSFC in the follow-up period (MSFC at baseline versus the 5th year, *P* = 0.02).

**Figure 2:**
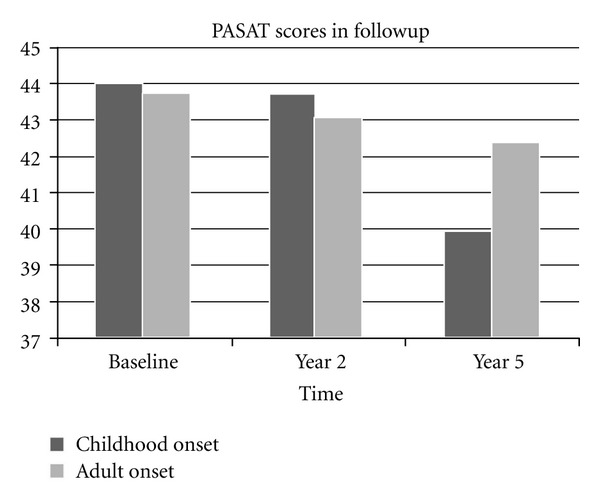
PASAT in the follow-up period (PASAT at baseline versus the 5th year, *P* = 0.008).

Although MSFC score seemed to be better in EOMS than AOMS group at baseline, it worsened more in childhood onset group than adult onset population at the end of the study (year 5). Difference in MSFC between baseline and at the end of the 5th year was significantly worse in EOMS population (*P* = 0.02, Figure 1). The most significant difference was found in Paced Auditory Serial Addition Test (PASAT) (*P* = 0.008, Figure 2). The mean number of correct responses in PASAT was 44.02 at baseline; it was decreased to 39.6 at the end of the study (*P* = 0.02). Mean 9-HPT time was increased from 21.70 at baseline to 23.02 at year 5, which was not statistically significant (*P* = 0.09). Mean 25-TWT score worsened from 6.12 at baseline to 6.94 at year 5 (*P* = 0.08). It was not statistically significant either.

**Figure 3:**
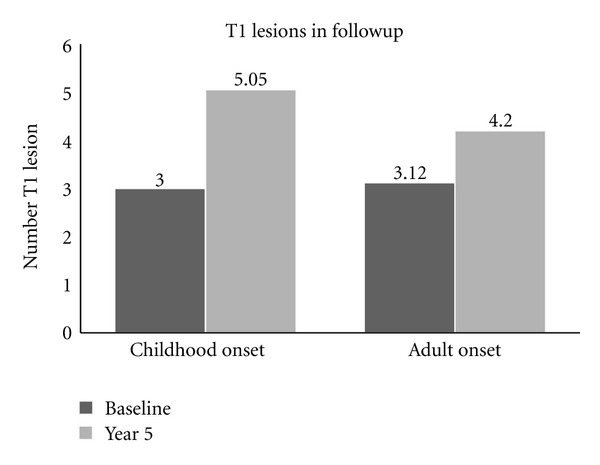
T1 hypointense lesions at baseline versus the 5th year, (*P* = 0.02).

There was no difference between childhood and adult onset MS, based on number of T2 hyperintense (24.94 and 27.8, resp.), Gd-enhanced (2.02 and 2.45, resp.), and T1 hypointense lesions (3.0 and 3.12, resp.) at baseline. Differences between baseline and end of the 5th year on the basis of T2 hyperintense lesions (ΔT2 lesions) and Gd-enhanced lesions (ΔGd-enhanced lesions) were not statistically significant, but there were significantly more T1 hypointense lesions in early onset MS than adult onset MS patients (Figure 3, *P* = 0.02).

### 3.1. Correlation Data

Correlation between the baseline overall MSFC and number of T2 lesions and Gd-enhanced lesions was moderately strong (*r* = −0.48, *P* = 0.032), but correlation between MSFC and number of T1 hypointense lesion (black hole) was stronger (*r* = −0.56, *P* = 0.026). When correlations performed on the basis of MSFC components, 9HPT and T25WT were found to be weakly correlated with number of T2 (*r* = 0.26 and *r* = 0.31, resp.) and T1 lesions (*r* = 0.30 and *r* = 0.28, resp.). Correlation between PASAT and number of T2 lesions was moderate (*r* = −0.42), but it was stronger when correlated with number of T1 lesions (*r* = −0.58). Changes in MSFC and MRI parameters between baseline and the fifth year were as follows. There was a weak correlation between ΔMSFC overall score and ΔT2 lesions (*r* = −0.18). There was also a weak correlation with ΔGd-enhanced lesions too. Correlation with ΔT1 was higher (*r* = −0.32). When correlation studies were performed with MSFC components, ΔT1 correlation was statistically significant with PASAT (*r* = 0.6), but it was extremely weak with T25WT and 9HPT (*r* = 0.13 and *r* = 0.2, resp.). There was no significant correlation between ΔGd-enhanced lesions and MSFC components.

## 4. Discussion

Multiple sclerosis is best recognized for its relapsing and remitting clinical course. In fact, in both children and adults, RRMS is the most common form, followed by the secondary and primary progressive forms. However, the prognosis of childhood onset MS remains controversial. There has been some evidence that patients with childhood onset MS have a slower accumulation of irreversible disability compared to patients with AOMS. But there is also conflicting evidence indicating that patients with childhood onset reach disability at a younger age than patients with adult onset [4, 7, 8, 14, 21]. Presented data is the first one regarding comparison of MRI and cognitive impairment in followup in childhood onset and adult onset MS.

There have been two opposite opinions regarding the impact on cognitive functioning in patients with childhood MS. It can be even more dramatic than adult onset cases, because the pathological process develops during early CNS myelinogenesis. Thus, it may negatively affect ongoing maturation of white matter pathways which can lead to neurodegeneration of neural networks involved in cognition. Amato et al. commented that this status might interfere with present and future academic achievements [11]. Conversely, brain plasticity and repair mechanisms may be more efficient in these ages, suggesting greater possibility of recovery and compensation. Available information on neuropsychological aspects of early onset MS remains rather poor, because there are a number of limitations, such as the lack of a suitable control group or the absence of a global measure of cognitive function. Regarding the pattern of cognitive impairment, prominent defects of verbal and visuospatial memory and complex attention were observed [11, 22, 23], which can be sufficiently screened by PASAT. In the present study, we compared a cohort of childhood and juvenile onset MS cases on their adulthood with a group of matched—on the basis of disease duration—AOMS controls to assess clinical status, especially cognitive impairment, and MRI parameters.

The definition EOMS used in previous studies has been variable. Some groups have used under the age of 18 and some others under 16. Although there has been seemed to have no significant differences, we used under age 16, because observations indicate that the closer the age to adulthood, the more the patients have similar features with adult onset MS [24, 25].

In the present study, we used MSFC in addition to EDSS for clinical assessment, because of EDSS's well-recognizeed disadvantages. Our results indicated that childhood and juvenile onset MS patients had a poorer prognosis than adult onset patients based on MSFC. Although there was no significant difference between EOMS and AOMS groups at baseline, MSFC significantly more worsened in childhood onset group than adult population at the end of the year 5. This difference seemed to be originated from PASAT scores. The mean number of correct responses was significantly less in EOMS patients than AOMS patients. Although 9HPT and T25WT scores were also worsened, they were not statistically significant, that is, trend only. Comparison of EDSS differences was also insignificant. As a most widely used screening test for cognition in MS, PASAT imposes high demands on the subject's working memory capacity, requiring controlled information processing (e.g., attention), visual memory, good auditory functioning, and calculating ability [26]. These features make PASAT a very convenient and practical tool to assess cognitive status in follow-up studies [19, 27], which was confirmed by our results.

Magnetic resonance imaging is the most sensitive paraclinical method supporting the diagnosis of multiple sclerosis. It detects areas of increased signal, predominantly in the white matter, on T2-weighted imaging and discloses abnormalities in 95% of patients with clinically definite MS. There have been no longitudinal MRI studies in EOMS to establish whether there is progressive atrophy of the brain or the appearance of black holes, which may reflect permanent disability. Although we did not perform atrophy assessment, our MRI data has novel evidence regarding prognosis in EOMS. Patients with early onset have significantly more T1 hypointense lesions (i.e., “black holes”) at the end of the year 5 followup comparing with AOMS patients. Difference in number of T1 lesions in year 5 followup was significantly more in early onset patients than adult onset. Moreover, significant correlations were established between PASAT and both T1 hypointense lesions at baseline and difference in number of T1 hypointense lesions at followup.

In conclusion, although we need some support with longer followup, our results indicate that early onset MS seems to have worse prognosis than adult onset MS on the basis of clinical manifestation, assessed by MSFC, cognitive impairment, and MRI parameters. Therefore, patients with early onset should be treated with immunomodulatory agents as early as possible to try to limit the process.

## Figures and Tables

**Table 1:** Comparison of demographic features of EOMS and AOMS at baseline.

	EOMS	AOMS	*P*
*N*	46	64	
Age at onset years ± SD	11.7 ± 3.28	29.6 ± 6.8	0.002
(range)	(8–16)	(19–41)	
Disease duration years ± SD	8.54 ± 6.65	7.97 ± 3.78	NS
Disease Course (%)			
RR	71.1	76.5	NS
SP	24.4	18.7	NS
PP	4.5	4.8	NS
EDSS at baseline	3.19 ± 2.71	3.46 ± 1.9	NS

EOMS: early onset multiple sclerosis, AOMS: adult onset multiple sclerosis, RR: relapsing remitting, SP: secondary progressive, PP: primary progressive, and EDSS: expanded disability status scale.

## References

[1] Hanefeld F, Bauer HJ, Christen HJ, Kruse B, Bruhn H, Frahm J (1991). Multiple sclerosis in childhood: report of 15 cases. *Brain and Development*.

[2] Sindern E, Haas J, Stark E, Wurster U (1992). Early onset MS under the age of 16: clinical and paraclinical features. *Acta Neurologica Scandinavica*.

[3] Cole GF, Stuart CA (1995). A long perspective on childhood multiple sclerosis. *Developmental Medicine and Child Neurology*.

[4] Simone IL, Carrara D, Tortorella C (2002). Course and prognosis in early-onset MS: comparison with adult-onset forms. *Neurology*.

[5] Duquette P, Murray TJ, Pleines J (1987). Multiple sclerosis in childhood: clinical profile in 125 patients. *Journal of Pediatrics*.

[6] Ghezzi A, Deplano V, Faroni J (1997). Multiple sclerosis in childhood: clinical features of 149 cases. *Multiple Sclerosis*.

[7] Boiko A, Vorobeychik G, Paty D (2002). Early onset multiple sclerosis: a longitudinal study. *Neurology*.

[8] Ozakbas S, Idiman E, Baklan B, Yulug B (2003). Childhood and juvenile onset multiple sclerosis: clinical and paraclinical features. *Brain and Development*.

[9] Hanefeld FA (1995). Characteristics of childhood multiple sclerosis. *The International MS Journal*.

[10] Bobholz JA, Rao SM (2003). Cognitive dysfunction in multiple sclerosis: a review of recent developments. *Current Opinion in Neurology*.

[11] Amato MP, Goretti B, Ghezzi A (2008). Cognitive and psychosocial features of childhood and juvenile MS. *Neurology*.

[12] Bye AME, Kendall B, Wilson J (1985). Multiple sclerosis in childhood: a new look. *Developmental Medicine and Child Neurology*.

[13] Dale RC, De Sousa C, Chong WK, Cox TCS, Harding B, Neville BGR (2000). Acute disseminated encephalomyelitis, multiphasic disseminated encephalomyelitis and multiple sclerosis in children. *Brain*.

[14] Ghezzi A, Pozzilli C, Liguori M (2002). Prospective study of multiple sclerosis with early onset. *Multiple Sclerosis*.

[15] McDonald WI, Compston A, Edan G (2001). Recommended diagnostic criteria for multiple sclerosis: guidelines from the International Panel on the Diagnosis of Multiple Sclerosis. *Annals of Neurology*.

[16] Lublin FD, Reingold SC (1996). Defining the clinical course of multiple sclerosis: results of an international survey. *Neurology*.

[17] Kurtzke JF (1983). Rating neurologic impairment in multiple sclerosis: an expanded disability status scale (EDSS). *Neurology*.

[18] Ozakbas S, Cagiran I, Ormeci B, Idiman E (2004). Correlations between multiple sclerosis functional composite, expanded disability status scale and health-related quality of life during and after treatment of relapses in patients with multiple sclerosis. *Journal of the Neurological Sciences*.

[19] Ozakbas S, Ormeci B, Idiman E (2005). Utilization of the multiple sclerosis functional composite in follow-up: relationship to disease phenotype, disability and treatment strategies. *Journal of the Neurological Sciences*.

[20] Fischer JS, Jak AJ, Knicker JE, Rudick RA (1999). *Administration and Scoring Manual for the Multiple Sclerosis Functional Composite Measure (MSFC)*.

[21] Renoux C, Vukusic S, Confavreux C (2008). The natural history of multiple sclerosis with childhood onset. *Clinical Neurology and Neurosurgery*.

[22] MacAllister WS, Belman AL, Milazzo M (2005). Cognitive functioning in children and adolescents with multiple sclerosis. *Neurology*.

[23] Banwell BL, Anderson PE (2005). The cognitive burden of multiple sclerosis in children. *Neurology*.

[24] Tardieu M, Mikaeloff Y (2004). Multiple sclerosis in children. *International MS Journal*.

[25] Achiron A, Garty B-Z, Menascu S (2012). Multiple sclerosis in Israeli children: incidence, and clinical, cerebrospinal fluid and magnetic resonance imaging findings. *Israel Medical Association Journal*.

[26] Sherman EMS, Strauss E, Spellacy F (1997). Validity of the Paced Auditory Serial Addition Test (PASAT) in adults referred for neuropsychological assessment after head injury. *Clinical Neuropsychologist*.

[27] Ozakbas S, Ormeci B, Kivircik Akdede BB, Alptekin K, Idiman E (2004). Utilization of the auditory consonant trigram test to screen for cognitive impairment in patients with multiple sclerosis: comparison with the paced auditory serial addition test. *Multiple Sclerosis*.

